# The Clinical Characteristics and Treatment of Cerebral Microarteriovenous Malformation Presenting with Intracerebral Hemorrhage: A Series of 13 Cases

**DOI:** 10.1155/2015/257153

**Published:** 2015-10-19

**Authors:** Jing-Fang Hong, Ying-Fang Song, Hai-Bing Liu, Zheng Liu, Shou-Sen Wang

**Affiliations:** ^1^Department of Neurosurgery, Fuzhou General Hospital of Nanjing Military Command, Dongfang Hospital, Xiamen University, Fuzhou 350025, China; ^2^Department of Pulmonary and Critical Care Medicine, Fuzhou General Hospital of Nanjing Military Command, Dongfang Hospital, Xiamen University, Fuzhou 350025, China

## Abstract

*Object*. The aim of this report was to explore the clinical presentation, radiological features, treatment methods, and outcome of micro-AVMs presenting with intracerebral hemorrhage. *Methods*. The clinical data, radiological features, treatment, and follow-up results for a consecutive series of 13 cases with micro-AVMs were retrospectively analyzed. *Results*. All 13 patients presented with intracerebral hemorrhage. Ten cases were confirmed by enhanced thin layer CT scanning and CTA, and the other 3 cases were confirmed by DSA. Treatment consisted of surgical removal in 10 cases, endovascular embolization in 1, and radiosurgery in 2. The modified GOS score was achieved in the third month after discharge: 10 cases were rated with 5 points (good recovery), 1 case was rated with 4 points (mild disability), and 2 cases were rated with 3 points (severe disability). During follow-up, No case of rebleeding was reported. *Conclusions*. Intracerebral hemorrhage is the main clinical manifestation of micro-AVMs. It is beneficial to find a tiny nidus of dense vessels located on hematoma wall on enhanced thin layer CT scanning for a clear diagnosis and to detect any abnormal feeding artery or venous drainage for an indirect diagnostic evidence. Resection is the main method of treatment for micro-AVMs.

## 1. Introduction

Arteriovenous malformation (AVM), composed of feeding artery, nidus, and venous drainage, is one of the most common vascular diseases of the cerebral hemorrhage. According to the clinical standard described by Yasargil, AVMs with a nidus smaller than or equal to 1 cm are called microarteriovenous malformations (micro-AVMs) [1]. In addition, the characteristics of small lesions and easy bleeding may pose a big challenge to the treatment and diagnosis of micro-AVMs. Meanwhile, there are only few reports and cases related to micro-AVMs to date, so the characteristics and treatment of the disease are still poorly understood. This study conducted a retrospective analysis from January 2008 to December 2013 and summarized the clinical data, radiological features, treatment, and outcome of these lesions in our department.

## 2. Patients and Methods

Thirteen cases of patients diagnosed as micro-AVMs were recruited in neurosurgery department of Fuzhou general hospital between January 2008 and December 2013. The patients were diagnosed as micro-AVMs with enhanced thin layer CT scanning, CT angiography (computed tomography angiography, CTA), cerebral digital subtraction angiography (DSA), or surgical exploration.

All patients underwent at least one enhanced thin layer CT scanning and CT angiography after being admitted to the hospital, and a following DSA examination would be conducted for the cases whose CTA finding was negative or questionable. Hematoma volume size was calculated according to formula *a* × *b* × *c*/2, where *a*, *b*, and *c* represent the maximal diameters of the hematoma in the 3 orthogonal planes. The original data of thin CT scanning for angiography was transferred to data postprocessing workstation and to obtain new 3D-CTA image of VR and MIP.

The corresponding treatment protocol in our series should be chosen according to the characteristics and position of micro-AVMs, hematoma volume size, and the clinical status of the patients. (1) Surgical removal is the main method of treatment for the superficial lesion, under the condition that the volume of hematoma is more than 40 mL and the patients began to complain of consciousness disturbance. It is preferred to conduct urgent surgery after CTA. In principle, it is necessary to remove some or all of the hematomas, after the identification of vascular malformation; the first thing is to cut off the feeding artery and then to remove the nidus, ensured with protecting the venous drainage. (2) Endovascular embolization is suitable for the lesion site located in the deeper brain and is not suitable for surgery removal. What is more, there is an existing optional feeding artery in such case. (3) Radiation therapy is suitable for the patient with the lesion located in functional area, or the case whose tortuous feeding artery is difficult to trace during microcatheter navigation.

Follow-up evaluation was conducted by means of the outpatient visits and telephone interviews. Follow-up evaluation content includes clinical examinations, CTA in all patients, and DSA in two cases. The mean duration of follow-up was 35 months (range 15–75 months) and final outcome was classified according to GOS standard score.

## 3. Results

Among the cases, there were 9 males (69.2%) and 4 females (30.8%), and the age ranged between 11 and 63 years with an average age of 32.6 years (Table 1). In terms of medical history, there was only one case with hypertension, and no cases were reported with any family history of AVMs. All cases were manifested as acute onset of cerebral haemorrhage with clinical symptoms including 13 cases of sudden headache, 6 cases of consciousness disturbance, 7 cases of hemiparesis, and one case of epileptic seizure.

**Figure 1:**
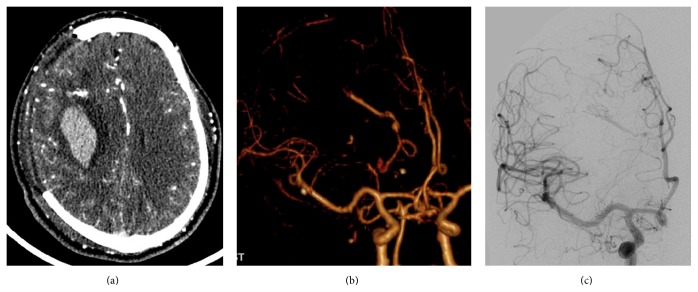
One case showed abnormal venous drainage in CTA. (a) Postoperative day 1 of decompressive craniectomy, enhanced thin layer CT scanning showing a tiny nidus of dense vessels in the front edge of hematoma. (b) VR reconstructed CTA images showing only abnormal venous drainage, no obvious nidus and feeding artery. (c) DSA confirmed micro-AVMs and feeding artery is a tiny branch of anterior cerebral arteries.

**Figure 2:**
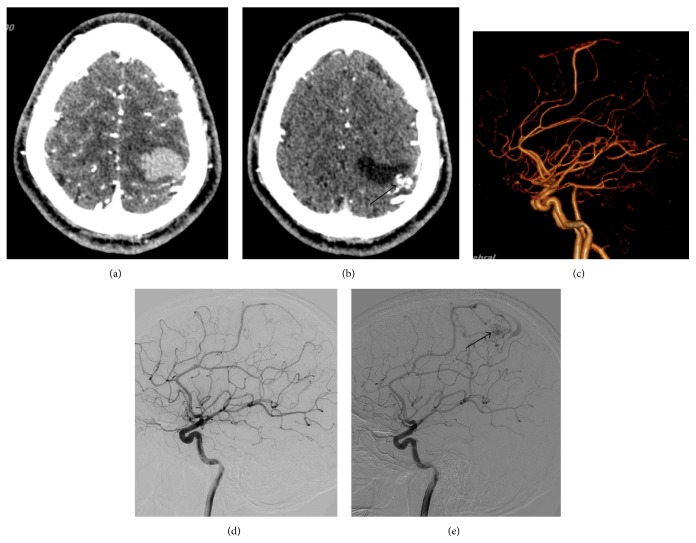
Different imaging findings of a micro-AVMs case before and after the hematoma absorption. (a) Enhanced thin layer CT scanning showed no obvious nidus on the day of the onset. (b) Enhanced thin layer CT scanning showed hematoma absorption and a tiny nidus of dense vessels was found after conservative treatment (arrow indicated). (c) Abnormal feeding artery without any nidus and venous drainage was found during initial CTA on the day of the onset. (d) Abnormal feeding artery without any nidus and venous drainage was found by DSA on the second day since onset. (e) Micro-AVMs, composed of feeding artery, nidus, and venous drainage, were clearly visible by means of DSA 8 months after conservative treatment (arrow indicated).

In evaluation of 13 cases with head CT scan, it was found that 12 patients had intracerebral hematoma, with 1 case of intraventricular hemorrhage. Among the cases, 4 hematomas were located in the temporoparietal lobe, 3 in the frontal lobe, 2 in the parietal lobe, 1 in the occipital lobe, 2 in the basal ganglia, and 1 in the left lateral ventricle. According to the formula, the hematoma was calculated to be 5~90 mL with an average value of 33 mL (Table 1). All 13 cases were examined with CTA, the enhanced thin layer CT scanning was applied to identify a tiny nidus of dense vessels located at hematoma wall in 10 cases (Table 1). After the application of VR reconstruction in postprocessing workstation, a whole micro-AVM composed of feeding artery, nidus, and venous drainage was identified in 7 cases, with the nidus invisible, but abnormal feeding artery was identified in 2 cases and abnormal venous drainage was found in 1 case. Three cases were reported to be negative by CTA. Five cases were examined with DSA and 4 cases were identified to be micro-AVMs. Among these five cases, only abnormal venous drainage was observed in one case with CTA (Case 13, Figures 1(a)–1(c)), and the following DSA examination indicated that it was a micro-AVM whose feeding artery was a tiny branch of anterior cerebral arteries. In another case, only abnormal feeding artery without any nidus and venous drainage was found during initial CTA and DSA examination. After 8 months, this case has been confirmed to be micro-AVMs by later CTA and DSA, and meanwhile feeding artery, nidus, and venous drainage were clearly visible (Case 10, Figures 2(a)–2(e)).

All 13 cases underwent definitive treatment, including surgical resection in 10 cases, endovascular embolization in 1 case, and X-knife radiotherapy in 2 cases. In the case of surgical operation, all cases were identified to be micro-AVMs during operation. The hematoma volume of 6 cases is found to be more than 40 mL, and upon complaints of symptoms like consciousness disturbance, an urgent brain hematoma removal and micro-AVMs resection were preferred. As a result, 4 cases were chosen for selective operation when a clear diagnosis has been established, including 2 cases under MRI neuronavigation in surgeries for micro-AVMs to localize the nidus and plan the craniotomy. One case of endovascular embolization to treat micro-AVMs located in arterial blood supply of the ventricle and additional two cases of radiation therapy were performed. In patients subjected to radiotherapy, 1 case was for eloquent area, which was treated with conservative treatment for the first 8 months, and afterwards, this case has been confirmed to be micro-AVMs by DSA, for which X-knife treatment was applied. Another case was reported in basal ganglia which is treated with X knife treatment for function protection purposes after the hematoma was absorbed. All cases were survived, except for 1 case which appeared with transient declined myodynamia, and the remaining 12 cases were recovered without any new complications after treatment. According to Glasgow outcome score, achieved in the third month after discharge, 10 cases (76.9%) were rated with 5 points (good recovery), 1 case (7.7%) was rated with 4 points (mild disability), and 2 cases (15.4%) were rated with 3 points (severe disability). During follow-up period, no case of rebleeding or development of new symptoms was reported.

## 4. Discussion

In 1987, Yasargil [1] first defined arteriovenous malformation whose nidus is smaller than or equal to 1 cm as micro-AVMs. Micro-AVMs are visible on angiography but in some cases only abnormal arterial blood supply or abnormal venous drainage can be found. The lesion should be identified from cryptic AVMs which can only be confirmed by postoperative histological examination [1] and are undetectable on angiography and at surgery. The current literatures about micro-AVMs are rare, so the true incidence of this disease is difficult to ascertain. Some previous studies [2–4] found that 8–10% of arteriovenous malformation has been confirmed to be micro-AVMs. Our studies accounted for about 8.2% of all AVM cases in our department during this period and were consistent with the previous reported data. Micro-AVMs may represent a possible source of major cerebral hemorrhage in young and middle-aged adults. In our study, the average age for this onset is 32.6 years. Except for a 63-year-old case with history of high blood pressure, no risk factors of cardiovascular diseases were found in these cases, which indicated that there might be a risk of micro-AVMs for young and middle-aged patients presenting with intracerebral hemorrhage without hypertension. The clinical manifestations of these 13 cases were intracranial hemorrhage, and micro-AVMs were reported to have very high risk of hemorrhaging [3–5], which is different from the other larger AVMs. As reported, only 38–70% of AVMs is associated with intracerebral hemorrhage, and it is generally believed that hemorrhaging risk is associated with the angioarchitecture of AVMs [6]. There are two reasons for the hemorrhage bleeding caused by micro-AVMs [4, 5]: the first reason is that the volume of lesion is small and flow volume is low, so that ischemia symptoms due to compressive or steal phenomena are not easy to identify; on the other hand, compared with large AVM with high-flow shunting, feeding pedicles of small AVM with low-flow shunting have higher pressure and a greater tendency of hemorrhaging. Bleeding occurs generally around the small bridging arteries that anchor the draining vein to the surrounding parenchyma, where arteriovenous fistula is easy to form and can cause venous outflow hindrance and hyperpressure [7]. Although micro-AVMs volume is small, the occurrence of bleeding can often lead to severe clinical symptoms [3, 5, 8]. The clinical manifestation depends on the location of hemorrhage and the volume of the hematoma, both of which call for an emergency surgery to remove the hematoma. In this study, the mean volume of hematoma is 33 mL, including 6 cases more than 40 mL, which leads to consciousness disturbance and cerebral hernia. In addition, there are 7 cases of limb paralysis, whose hematomas mostly located in the temporal and frontal cortex surface, so the appearance of bleeding may cause oppression to eloquent area. But after timely treatment, most patients' prognosis was good.

The diagnosis of micro-AVMs is difficult and challenging. In the case of middle-aged and young patients with large superficial hematoma, who had no medical history of high blood pressure, the suspicion of micro-AVMs was relatively high. Hino et al. [9] had reviewed the data of 137 patients with spontaneous subcortical hematoma, of which 41 cases were caused by specific vascular lesions. There are 4 cases where no lesions were detected on initial angiography but were later angiography visible. DSA is the best method for diagnosis for micro-AVMs, and in young patients whose conventional four-vessel cerebral DSA findings were negative or questionable, superselective angiography should be applied to suspicious vessels to increase the ability to reach a diagnosis of micro-AVMs according to the location of hematoma. Cellerini et al. [5] have conducted superselective angiography in 7 cases of suspected patients and confirmed the diagnosis of micro-AVMs. In their conclusion, for young patients with unexplainable bleeding, irrespective of negative or positive results of DSA, further selective microcatheter angiography was beneficial [5, 10]. Early hematoma compression may be one of the causes of negative DSA result and later DSA examination can be applied after hematoma absorption to improve the detection quality [2]. Also some scholars believed that the dynamic enhanced cranial MRA and the t2-weighted MRI images helped to find DSA negative patients [4]. In some cases, where patients developed the brain hernia caused by bleeding and were unable to conduct DSA or MR examination, CTA is expected to become a fast and convenient alternative option. In our study, all patients admitted to the hospital were examined initially by CTA, and the remaining negative CTA patients were examined by DSA. We assumed that the following three characteristics of CTA can help in diagnosis: (1) CTA after reconstruction is presented as micro-AVMs; (2) enhanced thin layer CT scanning can identify the tiny nidus of dense vessels around hematoma; (3) images after the reconstruction of CTA are presented as the abnormal feeding artery or venous drainage. One of the first two rules can be used as the criteria of diagnosis, whereas the third can be used as indirect evidence of diagnosis. In our study, among 13 cases of CTA examination, 10 cases showed the tiny nidus of dense vessels near hematoma, and after reconstruction of CTA, 7 cases were presented as micro-AVMs, 3 cases were found only with abnormal arterial blood supply, and only 1 case was found with abnormal venous drainage. Reconstruction of CTA images often present abnormal arterial or venous blood drainage, and the possible reasons are summarized as below: (1) small volume of micro-AVMs located in the superficial area which may lead to poor imaging after VR reconstruction; (2) the technician's lack of expertise to interpret the indirect signs such as abnormal arterial or venous blood drainage; (3) the hematoma compression, thrombosis, and vascular spasm, and so forth. Therefore, we believed that the diagnosis should not be too dependent on the reconstruction of CTA images, but more dependent on the reading of the original thin layer CT scanning before reconstruction. Through the reading of thin layer CT scanning, the tiny nidus of dense vessels around the hematoma can be found to indicate the location of nidus. For DSA and CTA negative patients, the possible reasons can be summarized as follows: the blood stream being slow, low-flow rate, pathological changes in thrombosis, arterial spasm and hematoma leading to pressure in vessels, and so forth. In the case of a patient with hematoma in eloquent area, DSA and CTA are found without any clear nidus, but with abnormal feeding artery. Eight months later, the CTA indicated a tiny nidus of dense vessels around the hematoma and DSA examination showed clear micro-AVMs (Figures 2(a)–2(e)). We finally assumed that, for patients in a critical condition, who were unable to conduct DSA, the high-quality CTA can serve as a good alternative and an abnormal arterial or venous blood drainage around the hematoma with tiny nidus of dense vessels can be used as a valuable indication for clinical diagnosis. To those who were diagnosed negative with initial CTA examination, we should conduct the DSA detection as soon as possible. For patients with negative result of initial CTA and DSA, from our own experience, repeated DSA examination 4 weeks later is required because at this time the hematoma will be liquefied or absorbed. For the highly suspected vascular disease cases with negative DSA and CTA, the thorough surgical exploration of the hematoma walls under the microscope is necessary.

Surgical resection, endovascular embolization, and radiotherapy are the three main treatment strategies for micro-AVMs. In our series, 10 patients underwent surgical resection: 1 was treated with embolization and 2 received radiotherapy. For the lesion on nonfunctional region of cortex, surgical resection is still the preferred treatment. Precise localization of the lesion is one of the main difficulties of in the operation. According to the preoperative imaging and the relationship between hematoma and lesions, neurosurgeons can largely determine the location of the lesions. In the case of venous drainage located in the superficial cortex, we can find lesions according to the arterialized venous drainage in a retrograde fashion; excision should follow the principle that feeding arteries was cut off firstly. If there no apparent lesion is found in arterial blood supply and venous drainage after craniotomy, we can gently remove hematoma and then search for the lesions according to the location of the hematoma. Lesions usually located in hematoma wall are not difficult to find. Neuronavigation technology or stereotactic technique can be beneficial to the surgery [4]. In this study, there was a case of micro-AVMs located in basal ganglia region and, for surgical operation of this deep micro-AVMs, we adopted neuronavigation with preoperative MRI to locate lesions which could help to minimize the postoperative neurologic damage. Endovascular embolization is highly recommended for treatment of lesions that are difficult to be accessed through surgical approach and with an obvious feeding arterial supply. In this study, we performed Onyx for a case of periventricular micro-AVMs, and the results were quite promising. Even though endovascular embolization of micro-AVMs is reported with good success rates, some studies largely relate it with potential bleeding risk [11]. Comparatively, Onyx's controllability is superior to other liquid embolic agents as it can avoid embolic agents entering normal blood vessels or venous drainage. We certainly believed that the treatment with Onyx may be safer, but there is no reported curative effect compared to NBCA. For cases that are not suitable for surgery and endovascular embolization, radiotherapy may be considered as a good alternative therapy, because the lesion is small and radiation is usually conducted after hematoma absorption. In this study, we performed X-knife treatment strategy with accuracy and effectiveness through enhanced CT positioning, and moreover, the patients had no complaints of rebleeding during the follow-up period.

## 5. Conclusions

Although micro-AVMs incidence is rare, significant clinical symptoms with hematoma and bleeding are very common. CTA can be used as a good method of emergency inspection because of its convenience and sensitivity. It is helpful to read carefully enhanced thin layer CT scanning, and abnormal feeding artery or venous drainage can be used as an indication for indirect diagnosis. In addition, surgical resection is still the preferred method of treatment for micro-AVMs, while radiation therapy and endovascular treatment can be used in case of deep lesions.

## Figures and Tables

**Table 1:** Overview of clinical features, diagnostic workup results, therapeutic methods, and outcome in the 13 patients with cerebral micro-AVMs.

Patient number/ age (y)/sex	Hematoma size (mL) & location	Enhanced thin layer CT scanning	CTA finding	DSA finding	Urgent surgery/treatment	Outcome (GOS)
1/11/M	20 Basal ganglia	Nidus	Micro-AVMs		No/surgery neuronavigation	5

2/31/F	10 Basal ganglia	Nidus	Abnormal feeding artery		No/radiotherapy	5

3/63/F	40 Parietal lobe	Nidus	Abnormal feeding artery		Yes/surgery	3

4/36/M	52 Temporoparietal lobe	Nidus	Micro-AVMs		Yes/surgery	5

5/18/M	27 Frontal lobe	Negative	Negative	Micro-AVMs	No/surgery	5

6/41/F	11 Frontal lobe	Nidus	Micro-AVMs		No/surgery neuronavigation	5

7/16/M	60 Temperoparietal lobe	Nidus	Micro-AVMs		Yes/surgery	5

8/15/M	16 Frontal lobe	Negative	Negative	Micro-AVMs	No/surgery	5

9/32/F	90 Temperoparietal lobe	Nidus	Micro-AVMs		Yes/surgery	4

10/24/M	10 Parietal lobe	Negative	Abnormal feeding artery	Abnormal feeding artery	No/radiotherapy	5

11/41/M	5 IVH	Nidus	Micro-AVMs	Micro-AVMs	No/endovascular embolization	5

12/61/M	48 Occipital lobe	Nidus	Micro-AVMs		Yes/surgery	5

13/35/M	50 Temperoparietal lobe	Nidus	Abnormal venous drainage	Micro-AVMs	Yes/surgery	3
